# *Porphyromonas gingivalis* Administration Induces Gestational Obesity, Alters Gene Expression in the Liver and Brown Adipose Tissue in Pregnant Mice, and Causes Underweight in Fetuses

**DOI:** 10.3389/fcimb.2021.745117

**Published:** 2022-01-13

**Authors:** Sumiko Yoshida, Masahiro Hatasa, Yujin Ohsugi, Yosuke Tsuchiya, Anhao Liu, Hiromi Niimi, Kazuki Morita, Tsuyoshi Shimohira, Naoki Sasaki, Shogo Maekawa, Takahiko Shiba, Tomomitsu Hirota, Tokuju Okano, Asuka Hirose, Rinko Ibi, Kanako Noritake, Yuki Tomiga, Hiroshi Nitta, Toshihiko Suzuki, Hirokazu Takahashi, Naoyuki Miyasaka, Takanori Iwata, Sayaka Katagiri

**Affiliations:** ^1^ Department of Periodontology, Graduate School of Medical and Dental Sciences, Tokyo Medical and Dental University (TMDU), Tokyo, Japan; ^2^ Oral Diagnosis and General Dentistry, Division of Clinical Dentistry, Tokyo Medical and Dental University Hospital, Tokyo Medical and Dental University (TMDU), Tokyo, Japan; ^3^ Division of Molecular Genetics, Research Center for Medical Sciences, The Jikei University School of Medicine, Tokyo, Japan; ^4^ Department of Bacterial Pathogenesis, Infection and Host Response, Graduate School of Medical and Dental Sciences, Tokyo Medical and Dental University (TMDU), Tokyo, Japan; ^5^ Comprehensive Reproductive Medicine, Regulation of Internal Environment and Reproduction, Systemic Organ Regulation, Graduate School, Tokyo Medical and Dental University (TMDU), Tokyo, Japan; ^6^ Division of Metabolism and Endocrinology, Faculty of Medicine, Saga University, Saga, Japan; ^7^ Liver Center, Saga University Hospital, Faculty of Medicine, Saga University, Saga, Japan

**Keywords:** periodontal disease, *Porphyromonas gingivalis*, small for gestational age, gestational obesity, liver, brown adipose tissue

## Abstract

Preventing adverse pregnancy outcomes is crucial for maternal and child health. Periodontal disease is a risk factor for many systemic diseases including adverse pregnancy outcomes, such as preterm birth and low birth weight. In addition, the administration of the periodontopathic bacterium *Porphyromonas gingivalis* exacerbates obesity, glucose tolerance, and hepatic steatosis and alters endocrine function in the brown adipose tissue (BAT). However, the effects of having periodontal disease during pregnancy remain unclear. Thus, this study investigates the effect of *P. gingivalis* administration on obesity, liver, and BAT during pregnancy. Sonicated *P. gingivalis* (Pg) or saline (Co) was injected intravenously and administered orally to pregnant C57BL/6J mice three times per week. Maternal body weight and fetal body weight on embryonic day (ED) 18 were evaluated. Microarray analysis and qPCR in the liver and BAT and hepatic and plasma triglyceride quantification were performed on dams at ED 18. The body weight of Pg dams was heavier than that of Co dams; however, the fetal body weight was decreased in the offspring of Pg dams. Microarray analysis revealed 254 and 53 differentially expressed genes in the liver and BAT, respectively. Gene set enrichment analysis exhibited the downregulation of fatty acid metabolism gene set in the liver and estrogen response early/late gene sets in the BAT, whereas inflammatory response and IL6/JAK/STAT3 signaling gene sets were upregulated both in the liver and BAT. The downregulation of expression levels of *Lpin1*, *Lpin2*, and *Lxra* in the liver, which are associated with triglyceride synthesis, and a decreasing trend in hepatic triglyceride of Pg dams were observed. *P. gingivalis* administration may alter lipid metabolism in the liver. Overall, the intravenous and oral administration of sonicated *P. gingivalis*-induced obesity and modified gene expression in the liver and BAT in pregnant mice and caused fetuses to be underweight.

## Introduction

Adverse pregnancy outcomes, such as preterm birth, small/large for gestational age, gestational diabetes, and hypertensive disorders, are serious health concerns, as they threaten the lives of both the mother and baby (Say et al., 2014; Liu et al., 2016). Particularly, preterm birth complications are the leading cause of death of infants under the age of five (Liu et al., 2016). Furthermore, obesity and severe liver dysfunction in pregnant mothers increase the risks of maternal and perinatal complications (Poston et al., 2016; Westbrook et al., 2016; Catalano and Shankar, 2017).

Periodontal disease is an infectious disease caused by periodontal bacteria; it triggers chronic inflammation and the destruction of tooth-supporting structures (Pihlstrom et al., 2005; Hajishengallis, 2015). Many systemic diseases, including diabetes and metabolic disorders, are epidemiologically associated with periodontal disease (Saito et al., 1998; Kocher et al., 2018; Genco and Borgnakke, 2020; Polak et al., 2020), and evidence supporting this claim has been reported in animal studies (Blasco-Baque et al., 2017; Komazaki et al., 2017; Polak et al., 2020; Watanabe et al., 2021). Systemic bacterial dissemination, swallowing bacteria, and systemic inflammation associated with periodontal disease are plausible pathways to systemic diseases (Hajishengallis, 2015; Olsen and Yamazaki, 2019).

A significant association between periodontal disease and preterm birth and low birth weight was found in multiple systematic reviews with meta-analysis (Vergnes and Sixou, 2007; Ide and Papapanou, 2013; Corbella et al., 2016; Vivares-Builes et al., 2018). In addition, periodontitis increases the risk for gestational diabetes (Abariga and Whitcomb, 2016). Administration of *Porphyromonas gingivalis*, a major periodontopathic bacterium, has been reported to cause obesity, impaired glucose tolerance, and aggravation of steatosis of the liver in mice after they were fed a high-fat diet (Sasaki et al., 2018). Furthermore, *P. gingivalis* administration altered endocrine function in the brown adipose tissue (BAT) (Hatasa et al., 2020). However, the effects of *P. gingivalis* on maternal obesity as well as liver and BAT functions under gestational conditions remain unclear.

Therefore, in the present study, we investigated the effects of the administration of *P. gingivalis* on pregnant mice, focusing on maternal obesity, fetal growth, and comprehensive gene expression in the liver and BAT.

## Materials and Methods

### Cultivation of *P. gingivalis*

*P. gingivalis* (ATCC 33277) was cultivated as described previously (Sasaki et al., 2018; Udagawa et al., 2018). Briefly, the bacteria were maintained on trypticase soy agar (Difco Laboratories, Detroit, MI, USA) supplemented with 10% defibrinated horse blood, hemin, and menadione at 37°C under anaerobic conditions. After 48 h, the bacteria were inoculated in trypticase soy broth and cultured at 37°C to the mid-log phase under anaerobic conditions. Subsequently, 10^9^ CFU/mL of the bacterial suspension was sonicated at an amplitude of 20 kHz for 5 min on ice using a Qsonica Q700 sonicator (Waken Btech, Kyoto, Japan). To confirm the viability of the sonicated bacteria suspension, it was cultivated to ensure that no colony was detected. The presence of endotoxin in the sonicated *P. gingivalis* suspension was confirmed in a previous study (Sasaki et al., 2018).

### Animals

**Figure 1:**
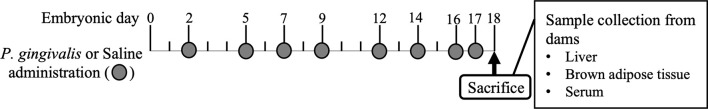
Experimental design and time schedule of the study.

Pregnant C57BL/6J mice (Embryonic day [ED] one; Sankyo Laboratory, Tokyo, Japan) were used in this study. The mice were provided *ad libitum* access to normal chow and water throughout the study and housed under standard conditions with a 12-h light/dark (light: 8:00 to 20:00) cycle. The mice were randomly divided into two groups: those administered with sonicated *P. gingivalis* suspension in the saline (Pg group, n = 10) and those receiving only saline (control [Co] group, n = 10). The administration was performed by both oral gavage and intravenous injection for each group. Sonicated *P. gingivalis* (10^8^ CFU) suspension in 100 μL of saline or 100 μL of saline only (control) was administered by two different methods, respectively. In total, 200 μL of saline containing sonicated *P. gingivalis* or 200 μL of only saline was administered to each mouse every round. Administration was performed three times a week from ED 2, followed by the evaluation of the body weight of the mice. The liver, BAT from interscapular fat, and plasma were harvested from the mothers on ED 18 after they had been fasted for 6 h. The body weight of the surviving fetuses and the survival rate of fetuses were evaluated. The experimental design and time schedule of the study are shown in the **Figure 1**. All protocols associated with animal use and euthanasia were reviewed and approved by the Animal Care Committee of the Experimental Animal Center at Tokyo Medical and Dental University (A2020-157A, 2021-107A).

### RNA Preparation

Extraction of total RNA from the liver and BAT samples (n = 10) were performed as described previously (Komazaki et al., 2017; Hatasa et al., 2021). In brief, the tissues were lysed and the aqueous layer containing RNA was separated using Trizol reagent (Invitrogen, Carlsbad, CA, USA). Then, total RNA was extracted from the layer using NucleoSpin^®^ RNA kit (TaKaRa Bio, Shiga, Japan).

### Microarray and Data Analysis

The quality of total RNA extracted from the liver and BAT were verified using an Agilent 2100 Bioanalyzer (Agilent Technologies, Santa Clara, CA). The Agilent Low Input Quick Amp Labeling kit (Agilent Technologies, Santa Clara, CA, USA) was used to generate complementary RNA (cRNA) from 200 ng of total RNA for single-color (Cy3) microarray analysis (n = 4), following the manufacturer’s instructions. Afterward, the cRNAs were hybridized onto an Agilent SurePrint G3 Unrestricted Gene Expression 8 × 60 K Microarray (Agilent Technologies). Fluorescence signals were detected using the Agilent Microarray Scanner System (Agilent Technologies). Raw microarray data were extracted using the Feature Extraction Software (ver. 11.0.1.1; Agilent Technologies).

### Quantitative Reverse-Transcription PCR Analysis

Reverse-transcription and real-time PCR were performed (n = 10) as described previously (Hatasa et al., 2021). Briefly, 500 ng of total RNA was reverse-transcribed to cDNA using the PrimeScriptTM RT Master Mix (TaKaRa Bio). Real-time PCR was performed using the Thermal Cycler Dice^®^ Real Time System II (TaKaRa Bio). PCR mixtures were prepared using TB Green Premix Ex TaqTM II (TaKaRa Bio). All procedures were performed following the manufacturer’s instructions. *Rn18s* was used as the reference gene for normalization. The PCR primers used in this study are listed in **Supplementary Table S1**.

### Hepatic Triglyceride Measurements

Triglyceride in the liver was measured as previously described (Le Marchand et al., 1973; Fujii et al., 2008; Komazaki et al., 2017; Sasaki et al., 2018). Briefly, the aliquots of liver lysates were added to a microcentrifuge tubes containing 37.5% KOH and heated at 70°C for 30 min. The tubes were placed in 55°C water bath overnight (n = 4). Subsequently, 50% ethanol was added, and the tubes were centrifuged. The supernatants were separated, treated with MgCl_2_, left on ice for 10 min, and then centrifuged again. The supernatants and a triglyceride standard (Sigma Aldrich [St. Louis, MO, USA]) were placed in a 96 well black plate with a clear flat bottom, and triglyceride levels were measured using a commercially available kit (Triglyceride quantification kit, Sigma Aldrich [St. Louis, MO, USA]). Preparation was performed by following the manufacturer’s instruction. Fluorescence intensity (λex = 540 nm/λem = 590 nm) was measured with a plate reader (BMG Labtech FLUOstar OPTIMA-6).

### Plasma Triglyceride Level Measurements

Blood samples with heparin were centrifuged and plasma was extracted. Commercially available kits were used to measure the plasma levels of triglycerides (n = 10) (Lab assay total triglyceride kit [290-63701], Wako [Osaka, Japan]) with a plate reader (Molecular devices SpectraMax ABS Plus). The measurement was carried out according to the manufacturer**’**s protocols.

### Statistical Analysis

Data distributions were analyzed using the Shapiro–Wilk test. The unpaired t-test was performed to compare the data from the two groups; differences with P < 0.05 were considered statistically significant. Data were analyzed using the R software (ver. 4.0.2). The microarray data were quantile-normalized and log_2_-transformed, and differentially expressed genes (DEGs) from these data were identified using R with the Limma Bioconductor package (ver. 3.40.6) (Ritchie et al., 2015). Benjamin and Hochberg’s false discovery rate (FDR) was applied for multiple testing. DEGs were defined in accordance with the following criteria: an FDR q < 0.1 and a |fold change| > 1.5. Overrepresentation enrichment analyses for the DEGs were performed using the WEB-based Gene SeT AnaLysis Toolkit (http://www.webgestalt.org) (Wang et al., 2013) and the Database for Annotation, Visualization, and Integrated Discovery (DAVID) (http://david.abcc.ncifcrf.gov/), along with the Gene Ontology (GO) and Kyoto encyclopedia of genes and genomes (KEGG) pathway databases. Gene set enrichment analysis (GSEA) (http://software.broadinstitute.org/gsea/index.jsp) (Subramanian et al., 2005) was performed using hallmark gene sets (Liberzon et al., 2015).

## Results

Significant weight gain and loss in the dams and fetuses, respectively, were observed and compared. The gene expression patterns in the liver and BATs associated with lipid synthesis and metabolism were studied to explain the weight gain and related impacts of *P. gingivalis* in dams.

### *P. gingivalis* Administration to Pregnant Mice Increased the Body Weight of Mothers but Decreased the Body Weight of Fetuses

**Figure 2:**
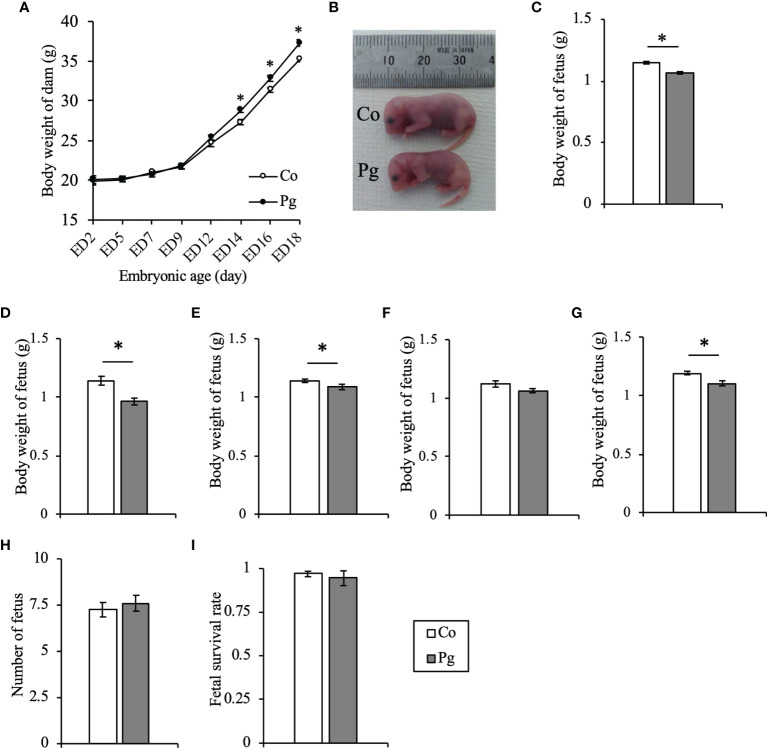
Sonicated *P. gingivalis* intravenous injection and oral administration induced maternal obesity and reduced body weight of fetuses. **(A)** Body weight of dams (n = 10, means ± SE, *P < 0.05). **(B)** Photograph of fetuses from Co and Pg dams at ED 18. **(C)** Body weight of fetuses at ED 18 (Co; n = 84, Pg; n = 76, means ± SE, *P < 0.05). **(D)** Body weight of fetuses at ED 18 from dams with five fetuses (Co; n = 5, Pg; n = 5, means ± SE, *P < 0.05). **(E)** Body weight of fetuses at ED 18 from dams with seven fetuses (Co; n = 55, Pg; n = 14, means ± SE, *P < 0.05). **(F)** Body weight of fetuses at ED 18 from dams with eight fetuses (Co; n = 8, Pg; n = 32, means ± SE, *P < 0.05). **(G)** Body weight of fetuses at ED 18 from dams with nine fetuses (Co; n = 9, Pg; n = 9, means ± SE, *P < 0.05). **(H)** Number of fetuses per dam (n = 10, means ± SE, *P < 0.05). **(I)** Fetal survival rate (n = 10, means ± SE, *P < 0.05).

The body weight of the Pg dams was increased significantly from ED 14 to ED 18 after both oral gavage and intravenous administration of sonicated *P. gingivalis* thrice per week. The differences in body weight between Co and Pg dams increased throughout the experimental period (**Figure 2A**). A significant decrease in the body weight of fetuses from the Pg dams was observed at ED 18 (**Figures 2B, C**). Comparing dams with the same number of fetuses, the body weight of fetuses from Pg dams significantly decreased in dams with five, seven, and nine pups (**Figures 2D, E, G**) and that tended to decrease in dams with eight pups (P = 0.069, **Figure 2F**). There were no significant differences in the number of fetuses (**Figure 2H**) obtained from, or the fetus survival rate (**Figure 2I**) of, the Co and Pg dams at ED 18.

### *P. gingivalis* Administration Altered the Gene Expression Patterns in the Liver of Dams

**Figure 3:**
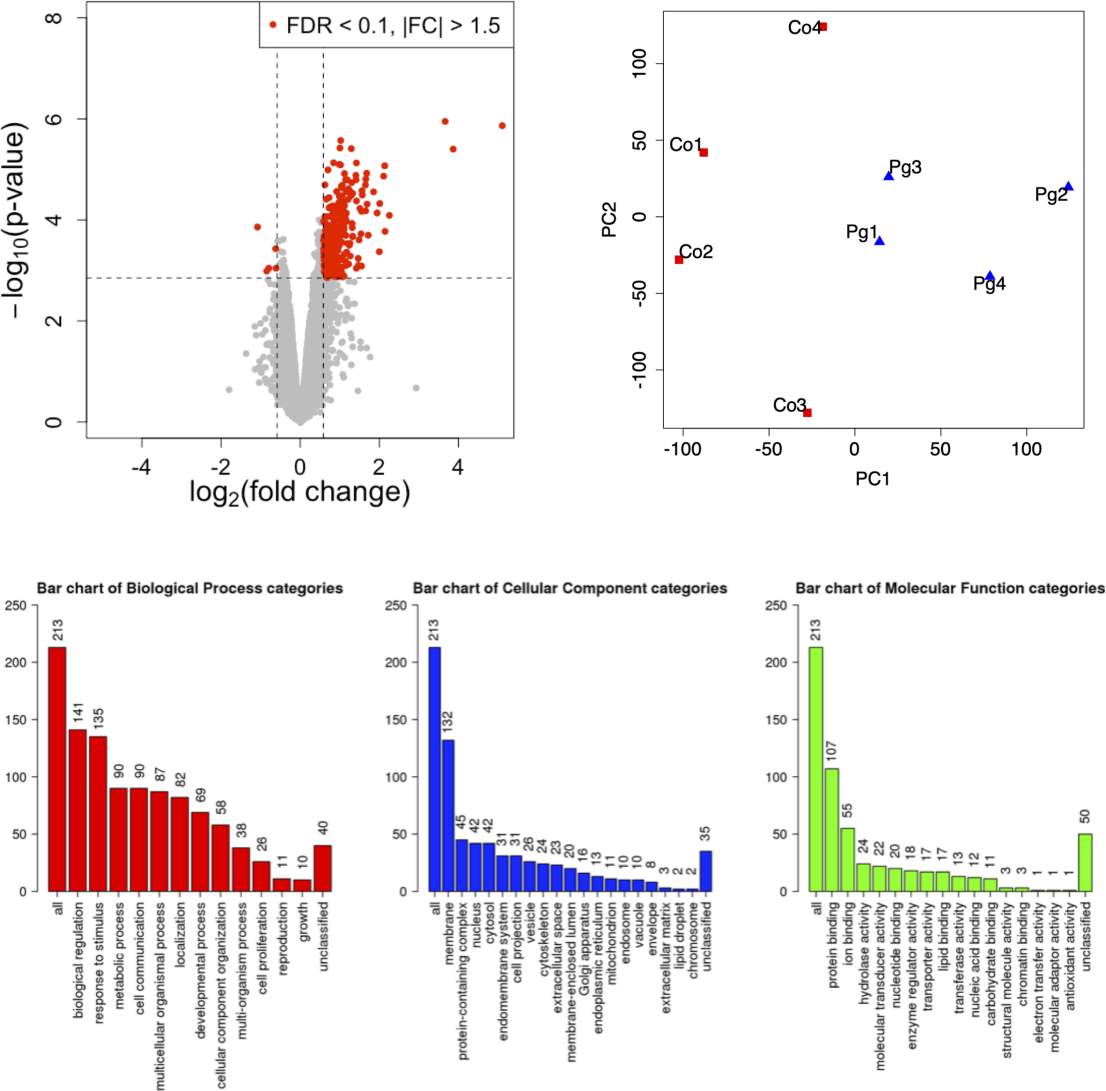
Microarray analysis in the liver between Co and Pg mice at ED 18 (n = 4). **(A)** Volcano plot; red plots show genes with FDR q < 0.1 and |fold change| > 1.5. **(B)** Principal component analysis. **(C)** Gene ontology in the upregulated DEGs.

To investigate gene expression in the liver following *P. gingivalis* administration during pregnancy, microarray analysis was performed to obtain a comprehensive overview of the gene expression profiles. All microarray data herein are available in the Gene Expression Omnibus (GEO) database (www.ncbi.nlm.nih.gov/geo) under GSE180189. As shown in **Figure 3A**, among 254 DEGs (|fold change| > 1.5 and q < 0.1), 248 genes were upregulated, and six genes were downregulated. The gene expression patterns differed substantially (**Figure 3B**). GO slim overviewed the ontology content in upregulated DEGs; interestingly, 42% of the upregulated DEGs with GO terms were classified as “metabolic process” in the biological process category (**Figure 3C**).

KEGG pathway analysis from upregulated DEGs was then performed; significantly enriched pathways in the liver (P < 0.05, Bonferroni q < 0.1) are listed in **Table 1**. Notably, “Natural killer cell-mediated cytotoxicity” and “B cell receptor signaling pathways” were included. There was no significantly enriched pathway associated with the downregulated DEGs in the liver.

**Table 1:** Kyoto encyclopedia of genes and genomes pathways in the upregulated differentially expressed genes in the liver of Pg dams at ED 18.

KEGG pathway	DEGs	p	Fold enrichment	Bonferroni
Osteoclast differentiation	*Btk, Fcgr1, Fcgr3, Tyrobp, Cyba, Lilra6, Lcp2, Ncf2, Pirb, Pira6, Pik3cd, plcg2, Sirpa, LOC100038947, Sirpb1b*	<0.001	9	<0.001
Natural killer cell-mediated cytotoxicity	*Cd48, Fcer1g, Rac2, Tyrobp, Itgb2, Lcp2, Pik3cd, Plcg2, Prkcb, Raet1e, Vav1*	<0.001	7.9	<0.001
Fc epsilon RI signaling pathway	*Btk, Fcer1g, Rac2, Lcp2, Pik3cd, Pla2g4a, Plcg2, Prkcb, Vav1*	<0.001	10	<0.001
Platelet activation	*Btk, Fcer1g, Rasgrp1, Adcy7, Apbb1ip, Lcp2, Pik3cd, Pla2g4a, Plcg2, Ptgs1, Tbxas1*	<0.001	6.3	0.0013
Leucocyte transendothelial migration	*Rac2, Cyba, Itgb2, Msn, Ncf2, Pik3cd, Plcg2, Prkcb, Rhoh, Vav1*	<0.001	6.4	0.004
Fc gamma R-mediated phagocytosis	*Fcgr1, Rac2, Was, Hck, Pik3cd, Plcg2, Prkcb, Vav1*	<0.001	7.2	0.019
B cell receptor signaling pathway	*Btk, Cd72, Cd79b, Rac2, Pik3cd, Plcg2, Vav1*	<0.001	7.5	0.051
*Staphylococcus aureus* infection	*Fcgr1, Fcgr3, C1qc, C1qa, C1qb, Itgb2*	<0.001	9	0.0081

The GO terms associated with the upregulated DEGs (P < 0.05 and Bonferroni q < 0.1) are listed in **Table 2**. Interestingly, many of these GO terms were related to the immune system, such as “Immune system process”, “Innate immune response”, “Adaptive immune response”, and “Acute-phase response”.

**Table 2:** Gene ontology terms in upregulated differentially expressed genes in the liver of Pg dams at ED 18.

GO term	p	Fold enrichment	Bonferroni
Immune system process	6.70E-19	8.3	6.30E-19
Innate immune response	3.60E-10	5.6	3.40E-07
Mast cell activation	1.00E-07	47.1	9.40E-05
Adaptive immune response	1.20E-07	8.8	1.10E-04
Positive regulation of type III hypersensitivity	3.60E-06	102.2	3.40E-03
Antigen processing and presentation of exogenous peptide antigen *via* MHC class I	8.90E-06	81.7	8.40E-03
Phagocytosis	1.10E-05	13.8	1.00E-02
Acute-phase response	2.10E-05	17.5	2.00E-02
Positive regulation of phagocytosis	1.00E-04	12.8	9.00E-02

**Figure 4:**
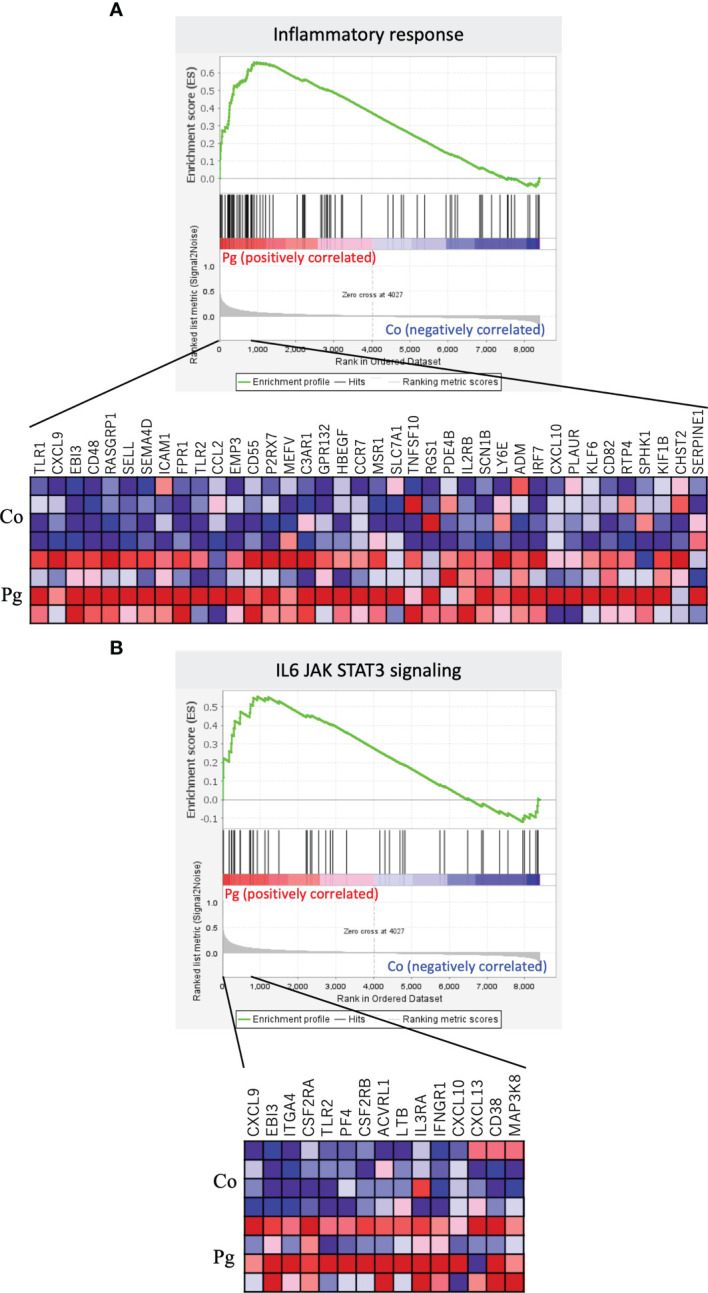
Notable gene sets enriched in the liver of Pg mice at ED 18 (n = 4). Gene sets about **(A)** Inflammatory response (NES = 1.97, q < 0.001) and **(B)** IL6/JAK/STAT3 signaling (NES = 1.53, q = 0.044). A heatmap provided illustrating gene expression levels for each gene in the core enrichment subset (blue: low, red: high).

**Figure 5:**
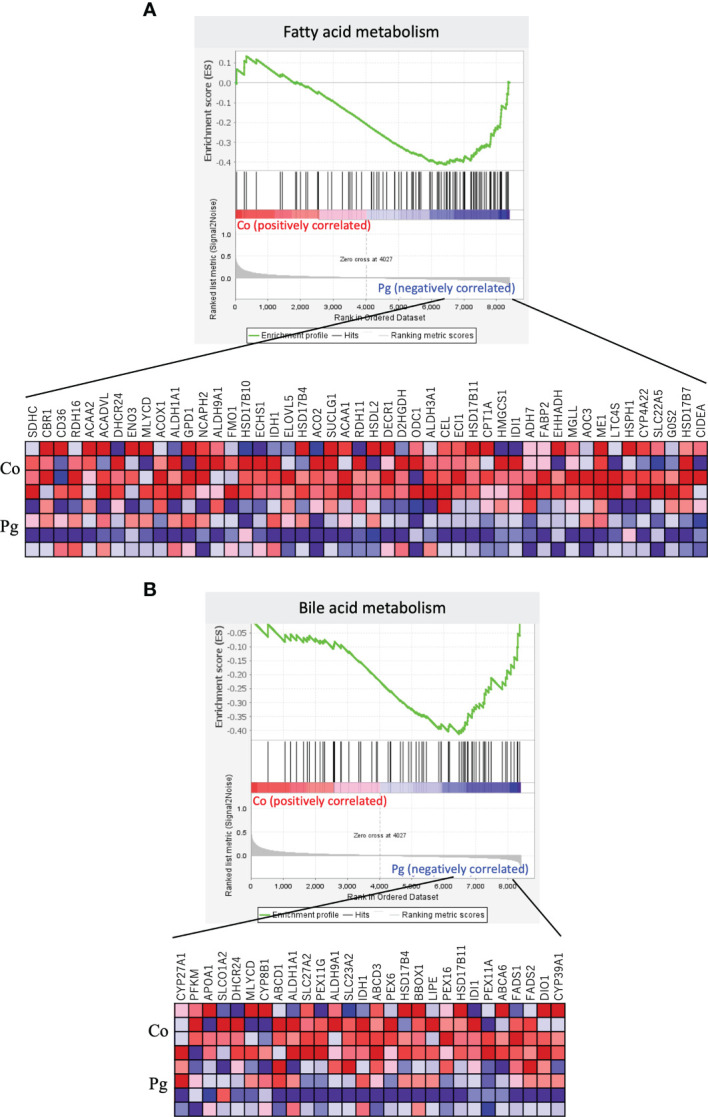
Notable gene sets downregulated in the liver of Pg mice at ED 18 (n = 4). Gene sets about **(A)** Fatty acid metabolism (NES = -1.74, q = 0.011) and **(B)** Bile acid metabolism (NES = -1.64, q = 0.027). A heatmap provided illustrating gene expression levels for each gene in the core enrichment subset (blue: low, red: high).

GSEA was performed using hallmark gene sets to evaluate differences in the mRNA expression levels of various genes in the livers of the Co and Pg dams. **Table 3** indicates the upregulated and downregulated gene sets with FDR q < 0.1. Several upregulated gene sets were related to inflammation, including inflammatory response set (**Figure 4A**, NES = 1.97, q < 0.001) and IL6/JAK/STAT3 signaling set (**Figure 4B**, NES = 1.53, q = 0.044). Interestingly, fatty acid metabolism gene set (**Figure 5A**, NES = -1.74, q = 0.011) and bile acid metabolism gene set (**Figure 5B**, NES = -1.64, q = 0.027) were downregulated.

**Table 3:** Gene set enrichment analysis with hallmark gene sets enriched and downregulated in the liver of Pg dams at ED 18.

Gene set	Size	NES	normal p-value	FDR q-value
Allograft rejection	97	2.23	<0.001	<0.001
Interferon gamma response	128	2	<0.001	<0.001
Inflammatory response	86	1.97	<0.001	<0.001
Interferon alpha response	64	1.91	<0.001	<0.001
Epithelial mesenchymal transition	102	1.87	<0.001	<0.001
Complement	121	1.8	<0.001	0.001
IL-2-STAT5 signaling	112	1.67	<0.001	0.009
KRAS signaling up	112	1.58	0.003	0.029
IL6/JAK/STAT3 signaling	46	1.53	0.02	0.044
Coagulation	86	1.44	0.029	0.098
Oxidative phosphorylation	123	-1.98	<0.001	0.002
Fatty acid metabolism	99	-1.74	<0.001	0.011
Bile acid metabolism	70	-1.64	0.007	0.027
Perioxisome	65	-1.61	<0.001	0.026
E2F targets	111	-1.51	<0.001	0.042
Spermatogenesis	49	-1.4	0.033	0.08
Myc targets v2	37	-1.35	0.069	0.092
Myc targets v1	112	-1.33	0.051	0.091

### *P. gingivalis* Administration Altered the Gene Expression Patterns in the BAT of Dams

**Figure 6:**
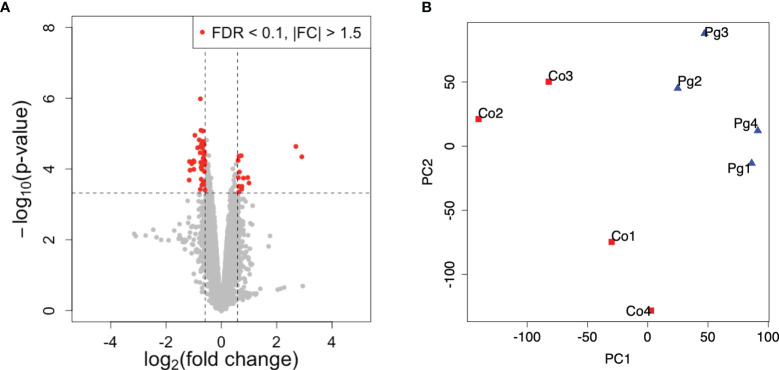
Microarray analysis in the brown adipose tissue between Co and Pg mice at ED 18 (n = 4). **(A)** Volcano plot; red plots show genes with FDR q < 0.1 and |fold change| > 1.5. **(B)** Principal component analysis.

Microarray analysis was also performed in the BAT from pregnant mice at ED 18. All microarray data presented herein are available in the GEO database under GSE180115. Only 53 DEGs (|fold change| > 1.5 and q < 0.1) were found (14 DEGs, upregulated; 39 DEGs, downregulated; **Figure 6A**). However, the gene expression patterns differed substantially (**Figure 6B**). The GO term “Lipid catabolic process” was found in downregulated DEGs showing a P < 0.05 and Bonferroni q < 0.1 (**Table 4**).

**Table 4:** Gene ontology terms in downregulated differentially expressed genes in BAT of Pg dams at ED 18.

GO term	p	Fold enrichment	Bonferroni
Lipid catabolic process	5.00E-04	24.6	9.60E-02

**Figure 7:**
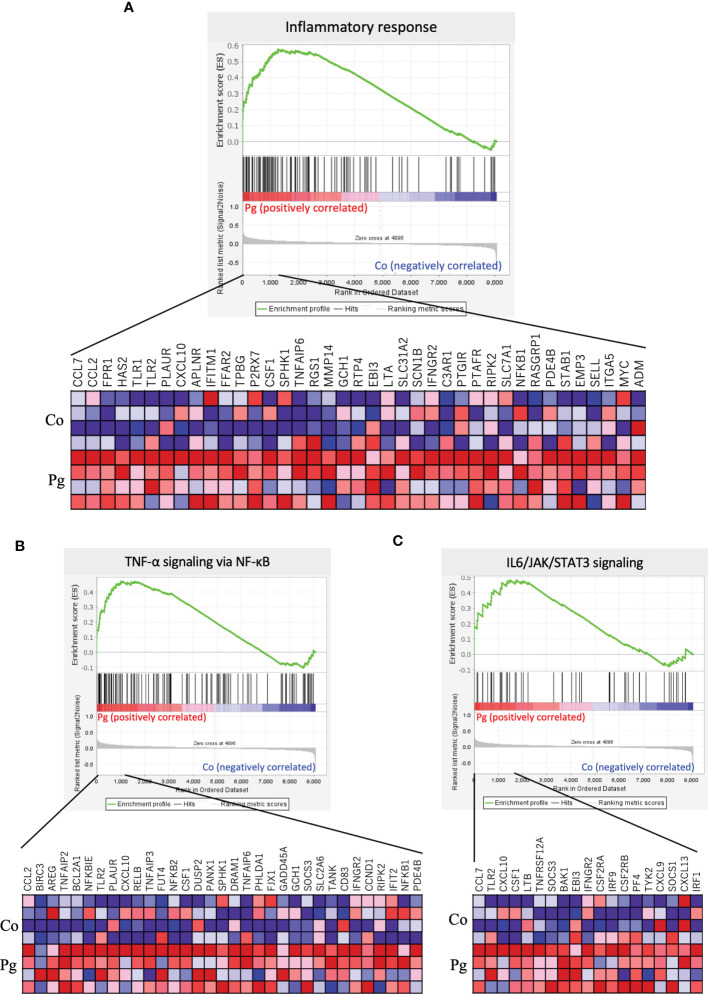
Notable gene sets enriched in the BAT of Pg mice at ED 18 (n = 4). Gene sets about **(A)** Inflammatory response (NES = 2.06, q < 0.001), **(B)** TNF-α signaling *via* NF-κB (NES = 1.70, q = 0.009), **(C)** IL6/JAK/STAT3 signaling (NES = 1.53, q = 0.023). A heatmap provided illustrating gene expression levels for each gene in the core enrichment subset (blue: low, red: high).

**Figure 8:**
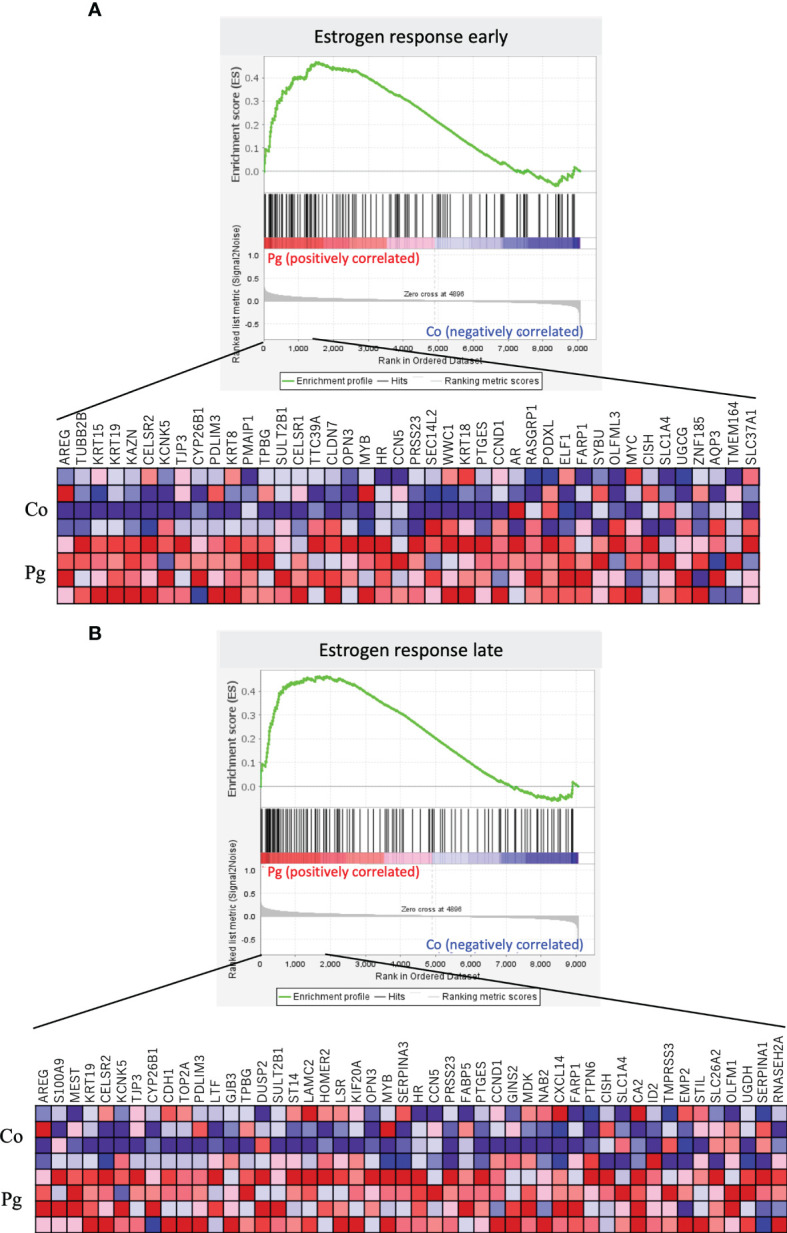
Notable gene sets enriched in the BAT of Pg mice at ED 18 (n = 4). Gene sets about **(A)** Estrogen response early (NES = 1.71, q = 0.009) and **(B)** Estrogen response late (NES = 1.71, q = 0.008). A heatmap provided illustrating gene expression levels for each gene in the core enrichment subset (blue: low, red: high).

GSEA was performed using hallmark gene sets to evaluate the differences between the mRNA expression patterns in the BAT of the Co and Pg dams. Enriched gene sets with FDR q < 0.1 are noted in **Table 5**. In accordance with the results of GSEA for the liver samples, the BAT of the Pg dams also showed the upregulation of gene sets associated with inflammation, including the inflammatory response gene set (**Figure 7A**, NES = 2.06, q < 0.001), the TNF-α signaling *via* NF-κB gene set (**Figure 7B**, NES = 1.70, q = 0.009), and the IL6/JAK/STAT3 signaling gene set (**Figure 7C**, NES = 1.53, q = 0.023). Surprisingly, the members of the estrogen response early/late gene sets were upregulated in the BAT of Pg dams (**Figure 8A**, NES = 1.71, q = 0.009; **Figure 8B**, NES = 1.71, q = 0.008).

**Table 5:** Gene set enrichment analysis with hallmark gene sets enriched and downregulated in BAT of Pg dams at ED 18.

Gene set	Size	NES	normal p-value	FDR q-value
Inflammatory response	96	2.06	<0.001	<0.001
E2F targets	122	1.99	<0.001	<0.001
Epithelial mesenchymal transition	117	1.87	<0.001	<0.001
G2M checkpoint	109	1.87	<0.001	<0.001
Interferon gamma response	133	1.8	<0.001	<0.001
Allograft rejection	101	1.79	<0.001	0.002
Estrogen response early	122	1.71	0.001	0.009
Estrogen response late	119	1.71	<0.001	0.008
TNF-α signaling *via* NF-κB	118	1.7	<0.001	0.009
Complement	116	1.66	0.001	0.01
IL6/JAK/STAT3 signaling	49	1.53	0.023	0.05
Apical junction	116	1.52	0.005	0.048
Interferon alpha response	66	1.51	0.022	0.049
Mitotic spindle	114	1.49	0.017	0.056
KRAS signaling up	120	1.46	0.02	0.074
Myogenesis	132	1.46	0.016	0.07
Myc targets	110	1.41	0.037	0.096
Oxidative phosphorylation	123	-1.72	0.012	0.03

### *P. gingivalis* Administration Downregulated the Transcript Levels of Genes Involved in Lipid Synthesis and Metabolism

**Figure 9:**
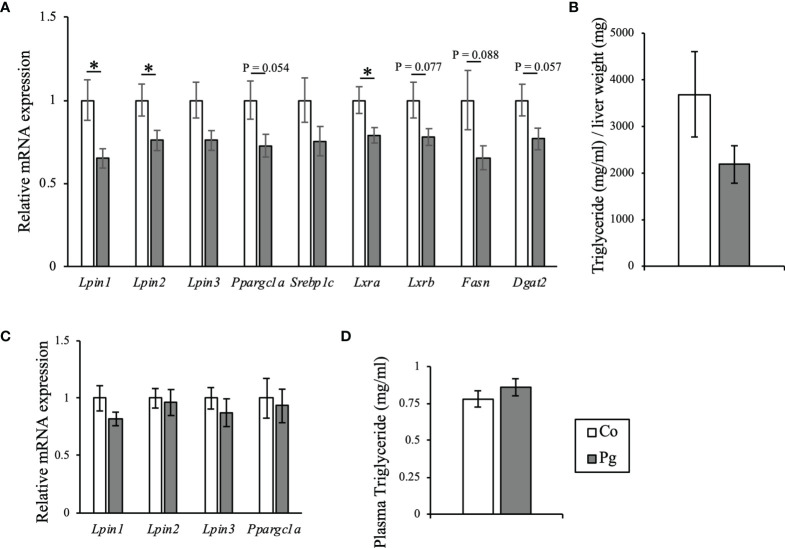
Evaluation of lipid metabolism in dams at ED 18. **(A)** Quantitative reverse-transcription PCR analysis in the liver. *Lpin1, Lpin2, Lpin3*, *Ppargc1a*, *Srebp1c*, *Lxra*, *Lxrb*, *Fasn*, and *Dgat2* expression (n = 10, means ± SE, *P < 0.05). **(B)** Hepatic triglyceride of dams at ED 18 (n = 4, means ± SE). **(C)** Quantitative reverse-transcription PCR analysis in BAT. *Lpin1, Lpin2, Lpin3* and *Ppargc1a* expression (n = 10, means ± SE). **(D)** Plasma triglyceride concentration of dams at ED 18 (n = 10, means ± SE, *P < 0.05).

Genes encoding lipins, which are involved in lipid synthesis *via* their phosphatidate phosphatase activity and act as transcriptional coactivators to regulate fatty acid oxidation, were among the DEGs that were downregulated in the liver of the Pg dams. Therefore, the transcript levels of lipin protein-encoding genes and other related genes were investigated. The expression levels of the Lipin 1 (*Lpin1*) and Lipin 2 genes (*Lpin2*) were significantly downregulated in the liver of Pg dams. Similarly, the expression of another lipin protein-encoding gene, *Lpin3*, was decreased in the liver of Pg dams. Moreover, the expression level of the peroxisome proliferator-activated receptor-gamma coactivator 1-alpha (*Ppargc1a*), which is the master regulator of mitochondrial biogenesis and induces lipin expression, decreased in the liver of Pg dams (p = 0.054). Furthermore, the administration of *P. gingivalis* significantly decreased the gene expression of *Lxra* in the liver, which is related to triglyceride synthesis. Other key genes for triglyceride synthesis (*Lxrb, Fasn*, and *Dgat2*) were downregulated in the liver of Pg dams (p = 0.077, p = 0.088, p = 0.057, respectively, **Figure 9A**). Liver triglyceride was evaluated for the validation of results from the microarray analysis and qPCR. Liver triglyceride from Pg dams tended to be decreased (**Figure 9B**). Contrastingly, in the BAT of Pg mice, there was no significant change in the transcript levels of the genes encoding lipins or peroxisome proliferator-activated receptor-gamma coactivator 1-alpha (**Figure 9C**). However, there were no significant differences between the concentrations of plasma triglycerides in the Co and Pg dams (**Figure 9D**).

## Discussion

Maternal periodontal disease has been reported to be associated with adverse pregnancy outcomes (Offenbacher et al., 1996; Vergnes and Sixou, 2007; Ide and Papapanou, 2013; Corbella et al., 2016; Figuero et al., 2020). A previous clinical study reported that threatened preterm labor and preterm birth were notably associated with the periodontal disease (Ye et al., 2013). In addition, another previous study have shown that sonicated *P. gingivalis* intravenous injection induced low birth weight in mice (Udagawa et al., 2018). However, the physiological changes in *P. gingivalis* administered dams remain unclear; ascertaining this was the target of the present study.

In this study, pregnant mice were subjected to both the intravenous injection and oral administration of sonicated *P. gingivalis* from ED 2. As periodontal disease features both endotoxiemia and the swallowing of periodontal bacteria (Hajishengallis, 2015), the administration of *P. gingivalis* was performed both orally and intravenously to mimic the clinical condition in this study. In addition, because the gestational period in mice is short, the effect of *P. gingivalis* administration had to be induced within the limited period of administration. Thus, administering both orally and intravenously is suitable for experimental design. The detection rate of *P. gingivalis* from plaque or saliva increased markedly in the mothers diagnosed with threatened preterm labor (Ye et al., 2013). In addition, the anti-*P. gingivalis* IgG titer in serum has been significantly higher in mothers with threatened preterm labor with small-for-gestational-age offspring than in those with threatened preterm labor with appropriate-for-gestational-age offspring (Ye et al., 2020).

In the present study, maternal obesity was also induced by the intravenous and oral administration of sonicated *P. gingivalis*. Excessive weight gain during pregnancy represents a risk for adverse pregnancy and neonatal outcomes (Stotland et al., 2006; Voerman et al., 2019). A previous study wherein endotoxemia was induced by administering *P. gingivalis* to pregnant mice *via* the dental pulp chamber did not report a change in the body weight of mothers (Ao et al., 2015). However, in an epidemiologic study, maternal overweight/obesity was reported to be associated with periodontitis and insufficient birth weight (Foratori-Junior et al., 2020). It is plausible that endotoxemia or oral administration of *P. gingivalis* induces obesity; this has been well reported in many previous studies. Endotoxemia induced by the administration of sonicated *P. gingivalis* after feeding mice with high-fat diets increased the body weight and visceral/subcutaneous fat mass, aggravated liver steatosis, and caused impaired glucose tolerance and insulin resistance (Sasaki et al., 2018). Furthermore, just a single intravenous injection of the sonicated *P. gingivalis* altered the endocrine activities of BAT (Hatasa et al., 2021). Oral administration of *P. gingivalis* also caused impaired glucose tolerance and insulin resistance (Arimatsu et al., 2014; Watanabe et al., 2021). Therefore, the maternal weight gain observed herein suggests that similar alterations in metabolism were induced during pregnancy after *P. gingivalis* administration. Thus, increased visceral and subcutaneous fat mass may be leading causes of weight gain.

Gestational weight gain is regarded as a high-risk factor of large-for-gestational-age or macrosomia (Goldstein et al., 2017; Goldstein et al., 2018). Interestingly, despite the gestational weight gain observed among the dams in this study, the fetal body weight was lesser after *P. gingivalis* administration. Previous studies on the maternal administration of *P. gingivalis* also reported a low birth weight and the birth of small-for-gestational-age pups (Ao et al., 2015; Udagawa et al., 2018); this is consistent with the results of the present study. All three studies, including this study, conducted multiple or continuous administration of P. *gingivalis* through almost the entire gestational period, which resulted in causing a lower fetal weight (Ao et al., 2015; Udagawa et al., 2018).

Microarray analysis in the liver and BAT was performed to evaluate the gene expression and compared these results with those from previous studies conducted using male mice (Sasaki et al., 2018; Hatasa et al., 2021). In the liver, the number of DEGs, particularly downregulated DEGs, were lesser than those in a previous study; this may have resulted from the shorter period of bacterial administration (16 days vs. 12 weeks) and the state of pregnancy in the current study. In the GO analysis of the present study, 42% of upregulated DEGs in the liver enriched with GO terms were classified as “metabolic process” in the biological process category; the DEGs from the male mice in the previous study showed a similar tendency (Sasaki et al., 2018). In the liver, pathways and GO terms related to the innate immune system were upregulated, such as the KEGG pathway “Natural killer cell-mediated cytotoxicity”, and the GO terms “Innate system process”, “Innate immune response”, and “Acute-phase response”. Natural killer cells, which are activated by dendritic cells, may be the source of interferon-γ, and may thus, be responsible for the *P. gingivalis*-specific IgG2 production in the gingival crevicular fluid and serum (Kikuchi et al., 2005). Interestingly, in the present study, the members from the interferon-gamma response gene set were also upregulated in the liver. Neutrophils are important for the maintenance of periodontal tissues. However, periodontal bacteria can interfere with the complement function and neutrophil-mediated killing in periodontal tissues (Hajishengallis, 2015). The results of the present study suggest that the innate immune system was disrupted by *P. gingivalis* administration. With regard to the adaptive immune system, the KEGG pathway “B cell receptor signaling pathway” and the GO term “Adaptive immune response” were enriched in the liver from Pg dams. As the plasma anti-*P. gingivalis* IgG antibody titer was increased in dams with *P. gingivalis*-induced endotoxemia (Udagawa et al., 2018), B cells may be activated by *P. gingivalis* in the present model. The members from the inflammatory response and IL6/JAK/STAT3 signaling gene sets were upregulated in the liver, suggesting that the inflammation of the liver is induced as a response to bacterial administration. Systemic inflammation caused by periodontitis is regarded as one of the possible mechanisms underlying adverse pregnancy outcomes (Madianos et al., 2013; Figuero et al., 2020). Periodontal disease may mediate systemic inflammatory pathologies (Hajishengallis, 2015) and eventually trigger pregnancy-related complications. Interestingly, members from the fatty acid metabolism gene set were downregulated in the liver; these were reported to be upregulated in the aforementioned previous study conducted using male mice (Sasaki et al., 2018). The concentration of maternal blood lipids and the blood glucose level are important factors that influence fetal growth (Kulkarni et al., 2013). Maternal lipid metabolism changes drastically during pregnancy, as highlighted by multiple changes in lipoprotein metabolism caused by elevated maternal insulin resistance and estrogen levels in the third trimester, which is critical to fetal growth (Herrera, 2002; Lain and Catalano, 2007; Barrett et al., 2014). In addition, members of the bile acid metabolism gene set were also upregulated in the liver. Maternal bile acid metabolism is important for fetal development (Brouwers et al., 2015; Pataia et al., 2017); it enables the absorption and metabolism of nutrients and fat-soluble vitamins and regulates the body’s sugar levels, lipid levels, energy metabolism, and endocrine and detoxification effects (Li and Chiang, 2014). As the disorder of bile acid metabolism can be induced by the injection of lipopolysaccharides from *Escherichia coli* in pregnant mice (Zhang et al., 2020), sonicated *P. gingivalis* injection may also affect fetal growth *via* altered bile acid metabolism.

Unlike the white adipose tissue, BAT contributes to thermogenesis by regulating the consumption of glucose and fatty acids (Nedergaard and Cannon, 2010). Interestingly, the loss of phenotype, whitening, and lipid accumulation of BAT are important for the maintenance of the gestational metabolic environment and fetal growth (McIlvride et al., 2017). In the GO analysis, members from the lipid catabolism gene set were downregulated in the BAT. This suggests that the alteration of the BAT phenotype may have been caused, which in turn, resulted in metabolic disorders in the mother and impaired fetal development. Furthermore, members of gene sets associated with responses to estrogen, the hormone that shows elevated levels in the third trimester and causes insulin tolerance and hyperglycemia (Baz et al., 2016), were upregulated in the BAT. Similar to the findings of a previous study that used male mice (Hatasa et al., 2020), in the present study, the BAT of pregnant mice also showed the upregulation of gene sets associated with inflammatory responses, such as the inflammatory response gene set, TNF-α signaling *via* NF-κB gene set, and IL6/JAK/STAT3 signaling gene set, suggesting that inflammation was induced in the BAT after *P. gingivalis* administration.

To detect the key genes responsible for altering maternal metabolism and weight gain, DEGs in both tissues were examined. *Lpin2*, which was listed as a DEG in the liver, plays a crucial role in lipid metabolism, along with the other lipin protein family genes *Lpin1* and *Lpin3* (Reue and Zhang, 2008). *Lpin1* mutation is responsible for lipodystrophy in fatty liver dystrophic mice (Péterfy et al., 2001). Lipin proteins function as lipid phosphatase enzymes to form diacylglycerol from phosphatidic acid for triglyceride synthesis (Han et al., 2006). Notably, *Lpin1* also plays a critical role as a transcriptional coactivator for *Ppargc1a*, and activates fatty acid oxidation and mitochondrial oxidative phosphorylation while suppressing lipogenesis and lipoprotein secretion. The upregulation of *Ppargc1a* expression activates *Lpin1*, and *Lpin1*, in turn, increases peroxisome proliferator-activated receptor alpha (PPARa) activity *via* the transcriptional activation of the PPARa gene and the direct coactivation of PPARa in cooperation with *Ppargc1a* and p300 (Finck et al., 2006). The expression levels of *Lpin1* and *Lpin2* were significantly downregulated in the liver of Pg dams, along with the downregulation of *Ppargc1a*; this implies that *P. gingivalis* administration may alter lipid homeostasis. Lipopolysaccharide and inflammatory cytokines reduce the expression of *Lpin1* (Lu et al., 2008; Meana et al., 2014); thus, the downregulation of *Lpin1* in this study may have been induced by the lipopolysaccharides derived from *P. gingivalis* and/or inflammation. Along with *Lpin 1* and *Lpin 2*, other key genes of triglyceride synthesis, *Lxra*, *Lxrb*, *Fasn*, and *Dgat2* were also downregulated in Pg administered dams. Furthermore, hepatic triglyceride tended to be decreased in Pg dams. A drastic change in maternal lipid metabolism is vital for fetal growth and development. Body fat accumulation is increased in the early stage of pregnancy. Conversely, during the third trimester, the catabolic process is accelerated, thereby elevating serum triglyceride cholesterol level (Herrera, 2002). The increase in plasma triglyceride corresponds to triglycerides synthesized in the liver (Wasfi et al., 1980). Moreover, the alteration of Liver X receptors signaling in the liver, which has key roles in the regulation of lipid metabolism, influences pregnancy adaptations in lipogenesis and protects against dysregulated fetoplacental lipid homeostasis (Nikolova et al., 2017). In the present study, although the plasma concentration of triglyceride of Pg dams was not significantly altered, significant downregulation of gene expression of triglyceride synthesis and decreasing trend of triglyceride in the liver of Pg dams were observed. The alteration of lipid metabolism in the liver induced by *P. gingivalis* administration may influence fetal growth and development.

However, in the BAT, there was no difference in the expression levels of any lipin genes or *Ppargc1a*. In addition, fewer DEGs were detected in the BAT than in the liver. These results suggest that the effects of *P. gingivalis* administration may be critical to the liver, which primarily performs detoxification and is connected directly to the portal vein.

In conclusion, the administration of *P. gingivalis* to pregnant mice causes gestational weight gain in dams and causes lower body weight in fetuses. Moreover, *P. gingivalis* administration altered the gene expression profiles in the liver and BAT of dams, suggesting the presence of inflammatory responses, immune responses, and altered metabolism. To the best of our knowledge, this is the first study reporting maternal obesity in pregnant mice administered with sonicated *P. gingivalis* and providing a comprehensive evaluation of gene expression in the liver and BAT of pregnant mice administered with *P. gingivalis*.

## Data Availability Statement

The original contributions presented in the study are included in the article/**Supplementary Material**. Further inquiries can be directed to the corresponding author.

## Ethics Statement

The animal study was reviewed and approved by the Animal Care Committee of the Experimental Animal Center at Tokyo Medical and Dental University.

## Author Contributions

SY performed most of the experiments and wrote the 1st draft of the manuscript. MH, YO, YTs, AL, HNii, KM, TShim, NS, SM, TShib, TH, TO, AH, RI, KN, YTo, HNit, TSu, HT, NM, and TI assisted in some studies and reviewed the manuscript. SK supervised all the studies and the writing of the manuscript. All authors contributed to the article and approved the submitted version.

## Funding

This work was supported by the Japan Society for the Promotion of Science (20H03863 to SK, 20K23020 to YTs, 19K24062 to NS, 19K18989 to SM, and 19K19016 to TShib).

## Conflict of Interest

The authors declare that the research was conducted in the absence of any commercial or financial relationships that could be construed as a potential conflict of interest.

## Publisher’s Note

All claims expressed in this article are solely those of the authors and do not necessarily represent those of their affiliated organizations, or those of the publisher, the editors and the reviewers. Any product that may be evaluated in this article, or claim that may be made by its manufacturer, is not guaranteed or endorsed by the publisher.
