# Supplementary material for: Porphyromonas gingivalis Administration Induces Gestational Obesity, Alters Gene Expression in the Liver and Brown Adipose Tissue in Pregnant Mice, and Causes Underweight in Fetuses
Source: Front Cell Infect Microbiol. 2022 Jan 13;11:745117. doi: 10.3389/fcimb.2021.745117 (PMC8792863; doi:10.3389/fcimb.2021.745117)
Supplement: Supplementary file 1 [file DataSheet_1.pdf]

Gene	Sense (5'-3')	Anti-sense (5'-3')
Lpin1	CTCCGCTCCCGAGAGAAAG	TCATGTGCAAATCCACGGACT
Lpin2	GAAGTGGCGGCTCTCTATTTC	AGAGGGTTACATCAGGCAAGT
Lpin3	TGGAATTGGGATGACAAGGT	CACTGCAAGTACCCCTTGGT
Ppargc1a	TATGGAGTGACATAGAGTGTGCT	CCACTTCAATCCACCCAGAAAG
Srebp1c	GGAGCCATGGATTGCACATT	GCTTCCAGAGAGGAGCCCAG
Lxra	CTCAATGCCTGATGTTTCTCCT	TCCAACCCTATCCCTAAAGCAA
Lxrb	ATGTCTTCCCCCACAAGTTCT	GACCACGATGTAGGCAGAGC
Fasn	GGAGGTGGTGATAGCCGGTAT	TGGGTAATCCATAGAGCCCAG
Dgat2	GCGCTACTTCCGAGACTACTT	GGGCCTTATGCCAGGAAACT
Rn18s	GTAACCCGTTGAACCCCATT	CCATCCAATCGGTAGTAGCG
